# Supplementary material for: Redesigned reporter gene for improved proton exchange-based molecular MRI contrast
Source: Sci Rep. 2020 Nov 26;10:20664. doi: 10.1038/s41598-020-77576-z (PMC7692519; doi:10.1038/s41598-020-77576-z)
Supplement: Supplementary file 1 — Supplementary Figures. [file 41598_2020_77576_MOESM1_ESM.pdf]

Supplementary Information: Redesigned Reporter Gene for Improved Proton Exchange-based Molecular MRI Contrast

Or Perlman[1], Hirotaka Ito[2], Assaf A. Gilad[3,4,5], Michael T. McMahon[6,7], E. Antonio Chiocca[2], Hiroshi Nakashima[2], Christian T. Farrar[1*]

[1] *Athinoula A. Martinos Center for Biomedical Imaging, Department of Radiology, Massachusetts General Hospital and Harvard Medical School, Charlestown, MA, USA*

[2] *Brigham and Women's Hospital and Harvard Medical School, Boston, MA, USA*

[3] *Department of Biomedical Engineering, Michigan State University, East Lansing, MI, USA*

[4] *The Institute of Quantitative Health Science and Engineering, Michigan State University, East Lansing, MI, USA*

[5] *Department of Radiology, Michigan State University, East Lansing, MI, USA*

[6] *F.M. Kirby Research Center for Functional Brain Imaging, Kennedy Krieger Institute, Baltimore, MD, USA*

[7] *Division of MR Research, The Russell H. Morgan Department of Radiology and Radiological Science, The Johns Hopkins University School of Medicine, Baltimore, MD, USA*

[*] Correspondence to: Christian T. Farrar, Athinoula A. Martinos Center for Biomedical Imaging, Department of Radiology, Massachusetts General Hospital, 149 13th Street, Suite 2301, Charlestown, MA, 02129, USA. Email: cfarrar@nmr.mgh.harvard.edu

```
                  CAAT                                        TATA          3´end of CMV promoter    Putative transcriptional start
761   CCCATTGACG CAAATGGGCG GTAGGCGTGT ACGGTGGGAG GTCTATATAA GCAGAGCTCT CTGGCTAACT AGAGAACCCA

                                     T7 promoter/priming site                         Hind III    Kpn I     BamH I
841   CTGCTTACTG GCTTATCGAA ATTAATACGA CTCACTATAG GGAGACCCAA GCTGGCTAGT TAAGCTTGGT ACCGAGCTCG

                         BstX I                                                    EcoR V       BstX I   Not I
921   GATCCACTAG TCCAGTGTGG TGGAATTGCC CTT PCR Product AAG GGC AAT TCT GCA GAT ATC CAG CAC AGT GGC
                            ACCTTAACGG GAA             TTC CCG TTA AGT
                                                       Lys Gly Asn Ser Ala Asp Ile Gln His Ser Gly

              Xho I      Xba I     Dra II  Apa I  Sac II  BstB I                  V5 epitope
987   GGC CGC TCG AGT CTA GAG GGC CCG CGG TTC GAA GGT AAG CCT ATC CCT AAC CCT CTC CTC GGT CTC
      Gly Arg Ser Ser Leu Glu Gly Pro Arg Phe Glu Gly Lys Pro Ile Pro Asn Pro Leu Leu Gly Leu

                          Age I       Polyhistidine region          Pme I                BGH Reverse
1053  GAT TCT ACG CGT ACC GGT CAT CAT CAC CAT CAC CAT TGA GTTTAAACCC GCTGATCAGC CTCGACTGTG
      Asp Ser Thr Arg Thr Gly His His His His His His ***

      priming site
1122  CCTTCTAGTT GCCAGCCATC TGTTGTTTGC CCCTCCCCCG TGCCTTCCTT GACCCTGGAA GGTGCCACTC CCACTGTCCT

        BGH polyadenylation signal
1202  TTCCTAATAA AATGAGGAAA TTGCATCGCA TTGTCTGAGT AGGTGTCATT CTATTCTGGG GGGTGGGGTG GGGCAGGAC
```

Supplementary Information Fig. S1. rdLRP DNA sequence.

a

b

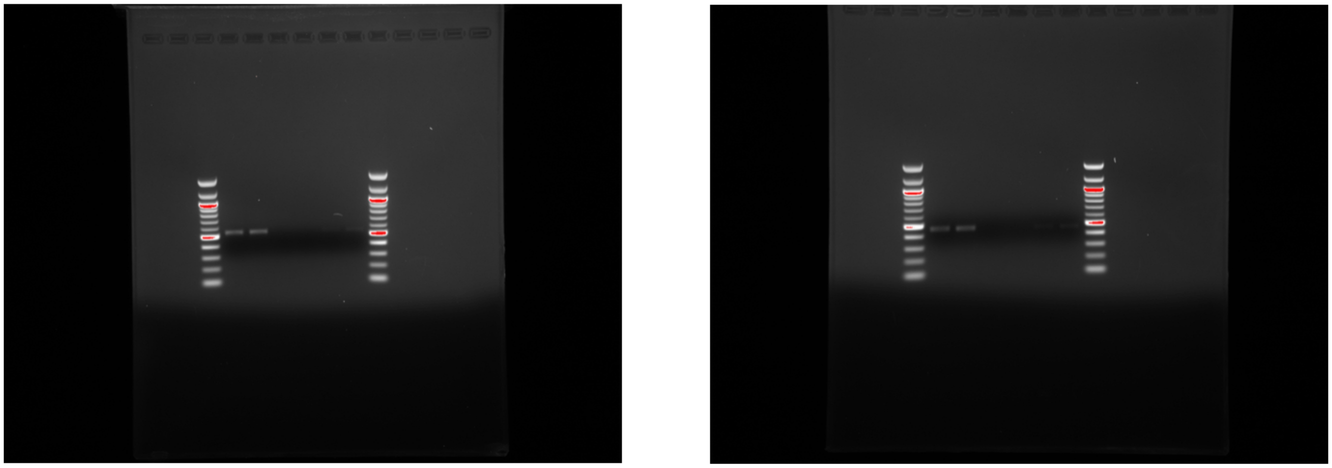

Supplementary Information Fig. S2. Raw gel images used for Fig. 2. RT-PCR of total mRNA from HEK293T cell lysates transfected with rdLRP, evaluating the BGH reverse tag (**a**) and the v5 epitope tag (**b**) sites. A single well-defined band is observed from cell lysates transfected with rdLRP, but not from control cell lysates. The base-pair lengths are consistent with expression of full-length rdLRP mRNA (b, 557 bp; c, 491 bp). 100 bp DNA ladder (N3231, New England BioLabs) was used as a scale marker. 1% agarose gels were run at 100V for 25 minutes. The photos were taken with ChemiDoc XRS+ System (Bio-Rad).

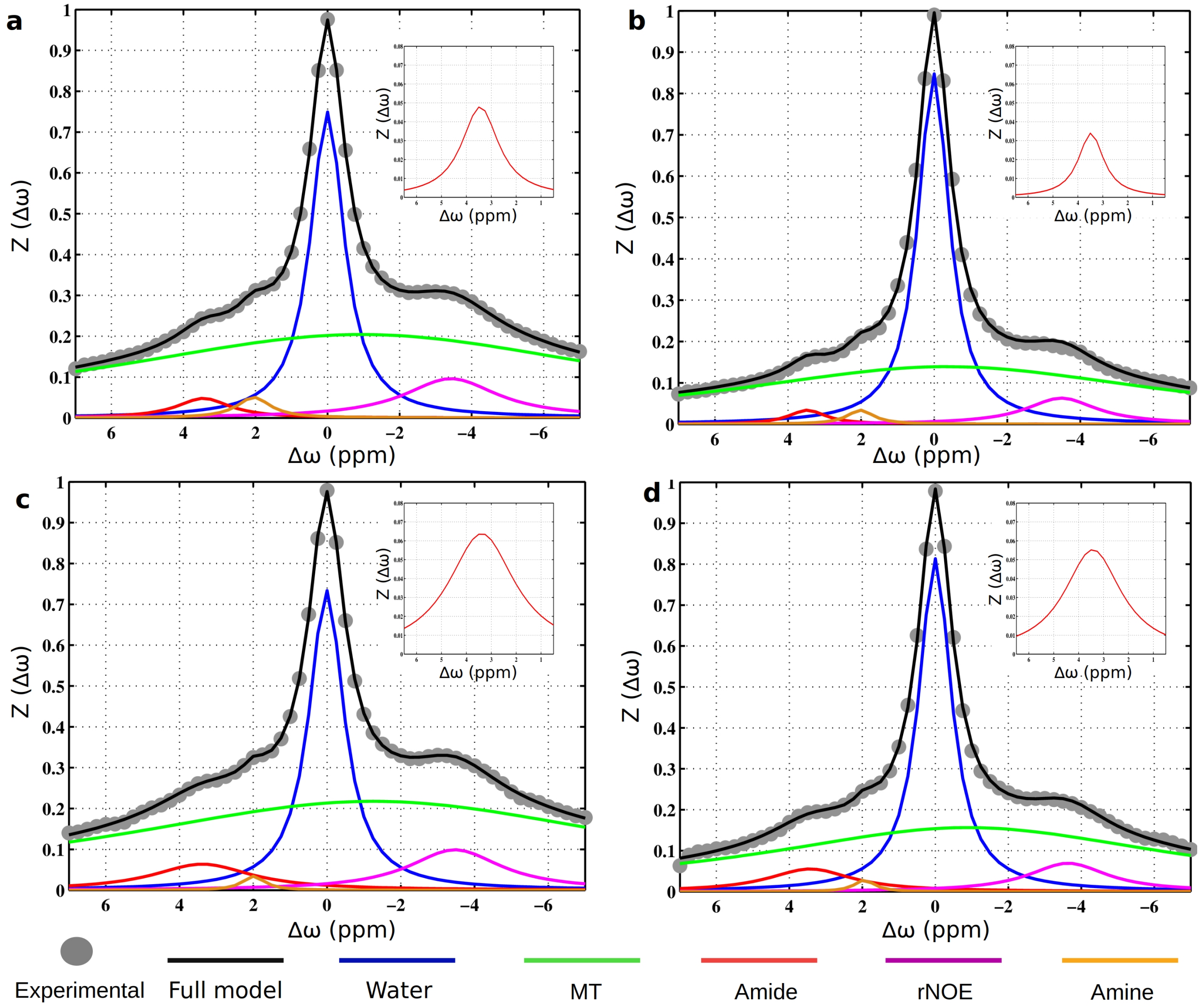

Supplementary Information Fig. S3. Extraction of the CEST amide proton amplitude *in vivo* using a Lorentzian fitting model. Each sub-figure represent the fitting of the experimentally measured z-spectra in the contralateral (left, **a**,**c**) and tumor (right, **b**,**d**) to a 5 pool model (water, semi-solid macro-molecules (MT), amide, aliphatic (rNOE), and amine), for the representative control (top row, **a-b**) and rdLRP (bottom row, **c-d**) mice, presented in Fig. 4. The insets provide a zoom-in display of the amide signal contribution.

Supplementary Information Table 1. Comparing the CEST contrast of hPRM1 and the rdLRP in HEK293 cell lysates.

	B_0(T)	Temp (°C)	pH	B_1(μT)	t_{sat}(ms)	Amine ΔMTR_{asym}(%)	Amide ΔMTR_{asym}(%)
hPRM1[24]	11.7	37	7.4	4.7	4000	0.63	0.37
rdLRP (this work)	4.7	25	7.5	3.6	5000	1.86	1.16
